# Assessment of efficacy and safety of advanced endoscopic irreversible electroporation catheter in the esophagus

**DOI:** 10.1038/s41598-023-33635-9

**Published:** 2023-05-16

**Authors:** Han Jo Jeon, Hyuk Soon Choi, Jae Min Lee, Eun Sun Kim, Bora Keum, Yoon Tae Jeen, Hong Sik Lee, Hoon Jai Chun, Seung Jeong, Hong Bae Kim, Jong Hyuk Kim

**Affiliations:** 1 Division of Gastroenterology and Hepatology, Department of Internal Medicine, Korea University College of Medicine, 73, Goryeodae-ro, Seongbuk-gu, Seoul, 02841 Republic of Korea; 2 Department of Biosystems and Biomaterials Science and Engineering, Seoul National University, Seoul, Republic of Korea; 3 Department of Veterinary Clinical Sciences, College of Veterinary Medicine, University of Minnesota, St. Paul, MN USA; 4 Masonic Cancer Center, University of Minnesota, Minneapolis, MN USA

**Keywords:** Oesophagogastroscopy, Gastroenterology, Gastrointestinal system, Oesophagus

## Abstract

Nonthermal irreversible electroporation (NTIRE) is emerging as a promising tissue ablation technique. However, maintaining irreversible electroporation (IRE) electrodes against displacement during strong esophageal spasms remains an obstacle. The present study aimed to evaluate the efficacy and safety of newly designed balloon-type endoscopic IRE catheters. Six pigs were randomly allocated to each catheter group, and each pig was subjected to four ablations at alternating voltages of 1500 V and 2000 V. Esophagogastroscopy was performed during the IRE. The ability of balloon-type catheters to execute complete IRE with 40 pulses was assessed. The success rate was higher for the balloon-type catheter than that for the basket-type (12/12 [100%] vs. 2/12 [16.7%], p < 0.001). Following gross inspection and histologic analysis of the 1500-V vs. 2000-V balloon-type catheter revealed a larger mucosal damage area (105.3 mm^2^ vs. 140.8 mm^2^, p = 0.004) and greater damage depth (476 μm vs. 900 μm, p = 0.02). Histopathology of the ablated tissue revealed separated epithelium, inflamed lamina propria, congested muscularis mucosa, necrotized submucosa, and disorganized muscularis propria. Balloon-type catheters demonstrated efficacy, achieving full electrical pulse sequences under NTIRE conditions, and a safe histological profile below 2000 V (1274 V/cm). Optimal electrical conditions and electrode arrays pose ongoing challenges.

## Introduction

The incidence of esophageal cancer (EC), one of the most aggressive cancers, has rapidly increased^1^. Although esophagectomy still plays a pivotal role in treating EC, recurrence has been reported in up to 50% of patients who have undergone surgery within 1–3 years^2^. According to a recent Surveillance, Epidemiology, and End Results (SEER) database analysis, the median overall survival time of patients with EC is 9 months, and the 5-year survival rate is 20%^3,4^. Due to the high local recurrence rate of EC and poor prognosis associated with EC distant metastases, a multidisciplinary plan for locally advanced EC (stages 2 and 3) that includes chemotherapy, radiation, or combination therapy is required to achieve a better survival rate^5^.

Regardless of treatment, early detection and elimination are vital factors that improve EC prognosis. Esophagectomy, a standard procedure for EC, has now been replaced by endoscopic submucosal dissection (ESD) for esophageal squamous cell neoplasia. ESD results in greater en bloc resection and is associated with shorter hospital stays and lower local recurrence rates, costs, and incidence of adverse events^6,7^. However, ESD is technically demanding and restricted to EC cases with a submucosal invasion < 200 µm, short segment (< 3 cm), and luminal circumferential lesion < 50%. Additionally, severe adverse events, such as stricture, dysphagia, and perforation, can occur frequently after ESD^8^.

Radiofrequency ablation (RFA) is a representative ablation therapy primarily used to destroy the esophageal squamous cell dysplasia and superficial esophageal squamous cell carcinoma^9,10^. It is well known that RFA is associated with lower stricture rates and shorter process times, as well as fewer learning curves^11^. One of the prevailing concerns with RFA is the potential for undertreatment and local tumor recurrence arising from incomplete ablation as a result of adjacent blood flow cooling down^12,13^. One way to overcome the heat-sink effect of RFA, however, is to use multi-electrode stereotactic RFA to improve local recurrence^14^. Nevertheless, the risks of incomplete ablation and tumor local recurrence post- RFA still remain^15^.

Irreversible electroporation (IRE) is a new therapeutic modality that has emerged as a minimally invasive treatment that applies electroporation to destroy the membranes of undesirable cells^16^. IRE has the benefit of being an ablation therapy that bypasses RFA by preserving surrounding structures, such as ducts and nerves, except for the target tissue. Nowadays, it is widely accepted that a new non-thermal ablative medical technique has favorable oncological outcomes for various solid organs^17^. However, little is known about its efficacy and safety in the esophagus.

We have previously investigated basket-type IRE catheters to ablate the esophageal mucosa using endoscopy. In our previous study, esophageal spasms with arching limited the overall delivery of IRE sequences^18^. Thus, researchers have become increasingly aware that successful ablation is largely dependent on overcoming intense esophageal contractions during stimulation. It was postulated that esophageal IRE interruptions may be modulated by altering the deployment tip of the balloon and that the balloon could potentially overcome this limitation imposed by the technique. Therefore, the current study aims to elucidate the efficacy and safety of balloon-type IRE catheters.

## Materials and methods

### Ethics statement

All research activities on animals were approved by the Institutional Animal Care and Use Committee of Korea University College of Medicine (no: KOREA-2021-0157) and performed in accordance with the relevant guidelines and regulations. Data acquisition, analysis, and interpretation were governed by the Animal Research Reporting of In Vivo Experiments (ARRIVE) guidelines.

### Animal preparation and experimental model

Six female YDL pigs (Yorkshire × Duroc × Landrace, XP-bio-Inc., Gyeonggi-do, Korea; average weight, 40 kg) were acclimatized for a 7-day quarantine period before use in the experiments. After premedication with atropine (0.02–0.04 mg/kg), the pig was first anesthetized with azaperone (2–8 mg/kg, intramuscular [IM]), xylazine (1 mg/kg, IM), and alfaxalone (4 mg/kg, IM) and isoflurane inhalation (1.5–2.0%). At the end of the study, the animals were euthanized using intravenous administration of potassium chloride (2 mmol/kg).

### Randomization and allocations

**Figure 1:**
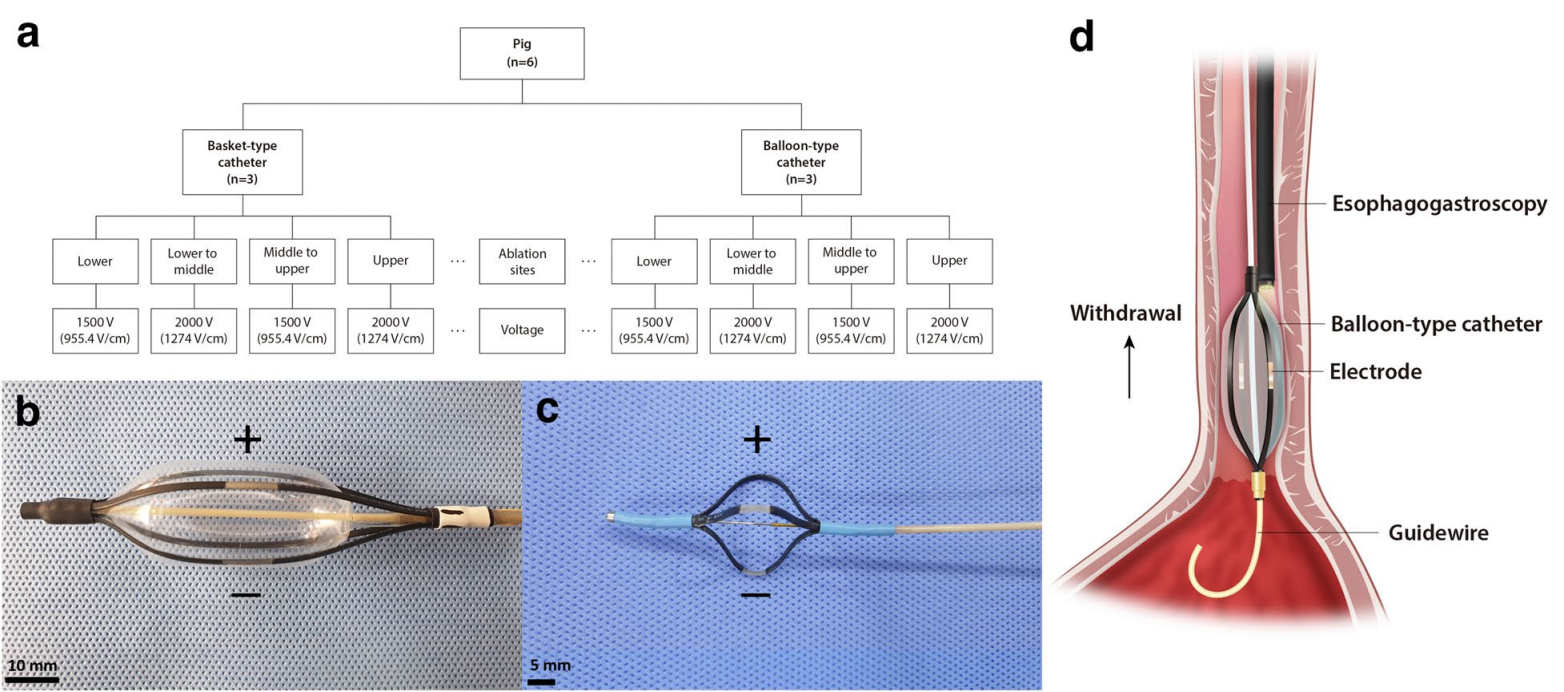
Study flow chart and illustration of endoscopic irreversible electroporation in the esophagus. (**a**) Flow chart of the IRE experiment; (**b**) Balloon-type catheter (nitinol electrode size: 1.5 mm, 10 mm, inter-electrode distances, 1.57 cm) placed over the guidewire (over-the-wire technique); (**c**) basket-type catheter (nitinol electrode size: 1.34 mm, 5 mm, inter-electrode distances, 2.09 cm) delivery through the working channel of the endoscope (through-the-scope technique); (**d**) Schematic design of irreversible electroporation experiments on pig esophagus.

The six pigs were randomly distributed into two groups: one using a balloon-type catheter and the other using a basket-type catheter (Fig. 1a). They were randomized using a computer-generated random number list prepared by an investigator with no experimental involvement in this study. Each group consisted of three pigs, and the esophageal mucosa of each one was ablated four times at alternating voltages of 1500 V and 2000 V to distribute an equal number of ablations. Each group underwent 12 electroporations (6 of 12 for 1500 V, 6 of 12 for 2000 V). Each ablation was considered an independent procedure. The tissue in the control group was obtained from the lowest part of the esophagus of each pig.

### Simulated electrical field and temperature distribution of the balloon catheter

A finite element analysis (FEA)-based model was adopted using COMSOL Multiphysics 5.6 (Stockholm, Sweden) to simulate the electrical field strength and thermal distribution of the balloon-type catheter electrode. Electrical and thermal properties were determined according to the esophagus properties database (https://itis.swiss/virtual-population/tissue-properties/database/, electrical conductivity 0.5 S/m, thermal conductivity 0.53 W/mK). A stationary and time-dependent study solver was adopted for the electric field strength and thermal distribution analyses.

### IRE equipment

#### IRE electrical protocol

Monopolar pulses were generated using BTX Gemini X2 (BTX^®^, Holliston, USA), delivered at a frequency of 10 Hz, width of 100 μs, an interval of 100 ms, number of pulses of 40, and a voltage of 1500 V or 2000 V. The generator was connected to the IRE catheter through a wire.

#### Balloon- and basket-type IRE catheter specifications

Two monopolar electrodes made of nitinol were designed for esophageal ablation (electrical conductivity, 1.219 S/m, thermal conductivity 0.18 W/mK). The IRE balloon-type catheter (EPO-B1; Standard Co. Ltd., Gyeonggi-do, Korea) is a transparent medical balloon composed of an inner tube that has a diameter of 1.5 mm made from polyether ether ketone (PEEK) and is configured to allow the guidewire to proceed (Fig. 1b). The catheter, 20 mm in diameter and 57 mm in length, was surrounded by four insulating tubes (polyolefin, Korea Ace Tech Co., Ltd., Korea), and two IRE electrodes, each measuring 1.5 mm and 10 mm and separated by approximately 1.57 cm, were mounted on the tubes. A basket-type IRE catheter (EPO-G2; The Standard Co. Ltd., Gyeonggi-do, Korea), identical to the one used in a previous study, was used^13^. The electrodes were sized 1.34 mm and 5 mm and separated by 2.09 cm (Fig. 1c).

#### Deployment process of IRE catheter

A 0.035-inch-wide guidewire was first positioned in the duodenum through the endoscopic working channel. The catheter was advanced along the esophagus while the guidewire was in place (over-the-wire technique) (Fig. 1d). Subsequently, the endoscope was reinserted, and the IRE balloon-type catheter was affixed 2 cm above the esophagogastric junction using endoscopic clipping (HX-610-135L, Olympus, Japan) to mark the lower margin of the balloon. Following the ablation of the lesion, the upper margin of the balloon was marked with a clip to be used later as the lower margin for subsequent ablation. Before ablation was applied, the inflated balloon was ensured to be in contact with the esophageal mucosa with the aid of contrast media (BONOREX^®^ 350 Inj, 30 mL, Dai Han Pharm, Korea) and C-arm radiographs (Cios Alpha^®^, Siemens Healthineers, Germany). Likewise, the basket-type catheter without a guidewire was evaluated similarly but without introducing the catheter through the channel (through-the-scope technique).

### Efficacy assessment of IRE catheters

“Success” of the procedure was defined as the delivery of all 40 pulses in one ablation without interruptions or technical issues, while “failure” was defined as the inability to deliver full sequences. The efficacy of the ablation procedure was measured by calculating the success rate, defined as the number of successful ablations out of 12 attempts. Another endoscopist recorded the number of successes as the two types of IRE catheters transferred the pulse. Further ablation of the same site was not permitted. Finally, an investigator who was not involved in the experimental procedures assessed whether or not the procedure was successful.

### Safety assessment of balloon-type catheter

#### Ablation area, depth, and histopathology

Esophageal tissue specimens obtained from euthanized swine were removed en bloc, followed by gross inspection. Each specimen was photographed, and the ablation outcomes—degree of erythema, erosion, and ulceration size—were configured using ImageJ software (IBM). Next, the specimens were fixed in 10% formalin for 1 day, excised at a thickness of 3 µm, and examined by hematoxylin and eosin (H&E) staining and terminal deoxynucleotidyl transferase (dUTP) nick end labeling (TUNEL) assay to analyze the depth of ablation. The tissue depth at which the most damaged cells were found was identified. The ablation area and depth were calculated as the average of the two lesions produced during esophageal ablation.

#### Current, impedance, and electrical energy

To measure the current between the two electrodes during IRE, a current probe (TCPA300; Tektronix, Beaverton, OR, USA) was coupled to an oscilloscope (TDA3044B; Tektronix, Beaverton, OR, USA) and clipped to an electrical wire from the pulse generator. Before and after the IRE, the impedance between the two electrodes was measured using a generator. The change in impedance of the esophageal tissue was calculated by subtracting the pre-ablation impedance from the post-ablation impedance. The electrical energy was calculated using the equation V^2^/R × t, where V is the applied voltage, R is the impedance, and t is the ablation time.

### Statistics

#### Minimal dataset

The differences in both groups were analyzed using a two-tailed G-power Fisher’s exact test at an alpha value of 0.05 and a power of 0.8. Each ablation was assumed to be independent of the other ablations. Considering the most appropriate ablation number to be 24, each group was allowed to undergo 12 ablation procedures, which denotes that 4 ablations were implemented in 1 swine esophagus. The minimum number of pigs that proved to be statistically significant in efficacy—the primary endpoint of this study—was 6.

#### Statistical analysis

Differences in the ablation success rate were evaluated using Fisher’s exact test on SPSS^®^ (version 24.0; IBM Corp, Armonk, NY, USA). Continuous variables are expressed as the median and interquartile range (IQR), while categorical variables are expressed as proportions. A non-parametric method using the Mann–Whitney U test was adopted to assess continuous variables in the 1500 V and 2000 V groups.

## Results

### Simulation results of balloon-type catheters on temperature and electrical field intensity distribution

**Figure 2:**
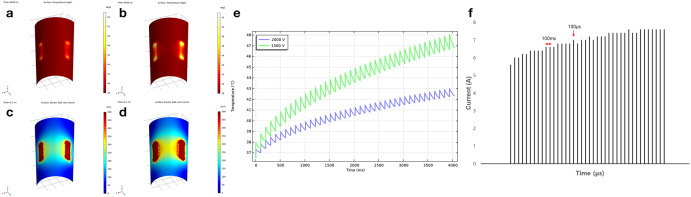
A two-dimensional virtual simulation of the temperature and electrical field intensity of the balloon-type catheter. (**a**) Temperature profile of the balloon-type irreversible electroporation catheter at 1500 V (955.4 V/cm); temperature close to the electrode plate is approximately 43 °C; (**b**) At 2000 V (1274 V/cm), the thermal condition of the electrode boundary is estimated to be about 48 °C; (**c**) Designed electrical field intensity at 1500 V (955.4 V/cm) showing a rectangular pattern; (**d**) Electric field distribution delineating the rectangular outline at 2000 V (1274 V/cm). The color bar represents the intensity of the electrical field; red scale, the highest value; and blue scale, the lowest value; (**e**) A graph illustrating the overall calculated increasing temperature response for 40 pulses in 4 s, which consisted of 100 ms of short pulse delivery with a 900-ms pause, leading to an increase and decrease in the temperature; (**f**) Elevation and saturation of electrical current during irreversible electroporation ablation at 2000 V (1274 V/cm).

The simulation results for the thermal distribution and temperature measurements after ablation are shown in Fig. 2. The edges of the electrodes in the graph turned a strong yellow, indicating an increase in the temperature. Before ablation, the initial temperature of the electrodes was set at 37 °C. Over time, the temperature fluctuated, ultimately reaching 43 °C (Fig. 2a) and 48 °C (Fig. 2b) 4 s after all 40 pulses were delivered at voltages of 1500 V and 2000 V, respectively. The temperature differences during ablation were estimated to be approximately 6 °C and 11 °C, respectively (Fig. 2c). A two-dimensional virtual electrical field intensity was calculated using the geometry to construct the model. At 1500 V, the electrical field created between the electrodes was hourglass-shaped, and its intensity was recorded at approximately 600 V/cm (Fig. 2d). In contrast, the overall electrical intensity was approximately 1000 V/cm at 2000 V, indicating rectangular morphology (Fig. 2e). The current during the full IRE sequence was measured. Forty pulses over 4 s were introduced into the esophageal epithelium from the IRE catheters. The oscilloscope detected current amplitudes and time changes during ablation (Fig. 2f). The current from the first pulse gradually increased and reached equilibrium.

### Endoscopic and fluoroscopic view during catheter ablation

#### Success rate of ablation with basket- and balloon-type catheters

**Figure 3:**
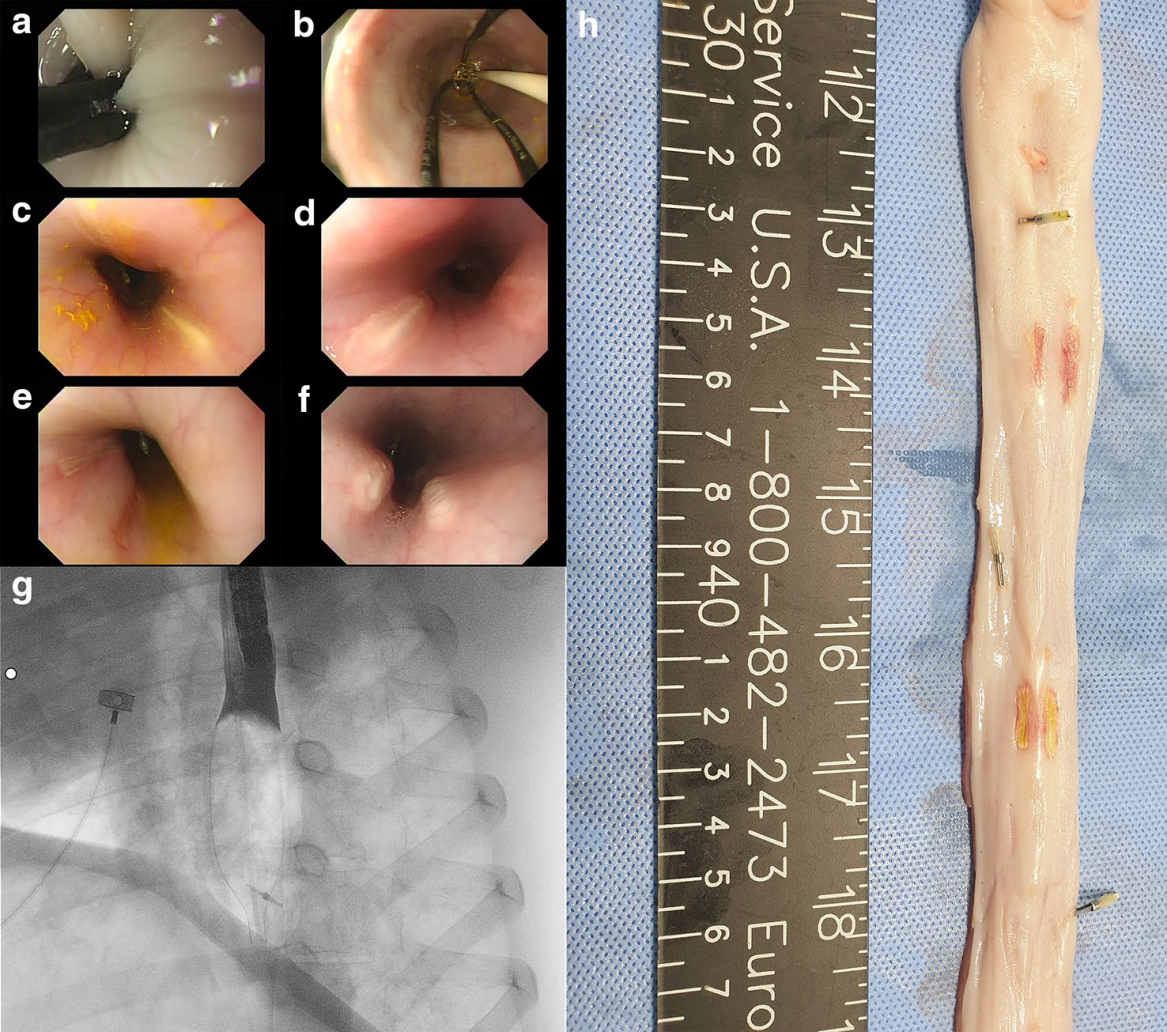
Endoscopic view of irreversible electroporation ablation (IRE). (**a**) Using a basket-type irreversible electroporation catheter, intense muscle contraction is noted, which prevents endoscopic full sequence delivery of IRE and interrupts ablation; (**b**) Balloon-type irreversible electroporation catheter endures the esophageal spasm and also allows the endoscopists to observe the IRE process through the transparent balloon during ablation; (**c**) Esophageal injury post-ablation with 1500 V immediately shows a pale mucosal surface; (**d**) With a 2000 V stimulus, endoscopy immediately displays a whitish mucosal surface with a slightly erythematous change at the periphery; (**e**) Within 24 h after ablation with 1500 V, the esophageal mucosal layer exhibits patterns of edema; (**f**) Mucosal swelling post-ablation (24 h) with 2000 V; (**g**) A radiologic image reveals a dilated balloon with contrast medium stasis; (**h**) Inverted esophageal tissue showing endoscopically placed clips spaced regularly within the IRE-ablated mucosa.

The esophageal muscle spasms produced during ablation elicited a narrowing of the inter-electrode distance, which was impeded by arching (Fig. 3a). Most ablations using basket-type catheters failed to achieve a complete delivery; in contrast, the balloon-type IRE catheter withstood strong esophageal contractions and successfully delivered all sequences. Furthermore, the transparency of the balloon offered a clear endoscopic view during ablation (Fig. 3b).

#### Endoscopic comparison of 1500 and 2000 V electrical conditions with a balloon-type catheter

Only the esophageal mucosa in contact with the two rectangular electrodes of the IRE catheter showed mucosal alterations, while no changes were observed in the tissue between the electrodes, suggesting that non-contiguous ablation was achieved. The ablation zone margin at 1500 V exhibited a whitish mucosal alteration immediately after the stimulus (Fig. 3c), followed by prominent edema, and a remarkable whitish change after 24 h (Fig. 3d). Following ablation at 2000 V, the esophageal mucosa showed a slight central depression with whitish mucosal changes and marginal erythema (Fig. 3e). The epithelium became whitish and swollen after 24 h (Fig. 3f). The overall endoscopic findings of 2000 V ablation revealed a wider mucosal change and a more swollen surface than those of 1500 V ablation.

#### Fluoroscopic examination during catheter ablation

After clipping the lowest margin of the balloon to ensure accurate contact with the target lesion, the balloon was tightly deployed into the lumen of the esophageal wall, as confirmed on C-arm radiographs. Stasis of the contrast medium over the inflated balloon indicated complete esophageal lumen obstruction by the balloon (Fig. 3g). then, a gross examination of the ablated esophagus revealed ablation and clipping at regular intervals (Fig. 3h).

### Safety assessment of balloon-type catheters

#### Histologic evaluation of post-IRE esophagus tissue; 1500 V vs 2000 V

**Figure 4:**
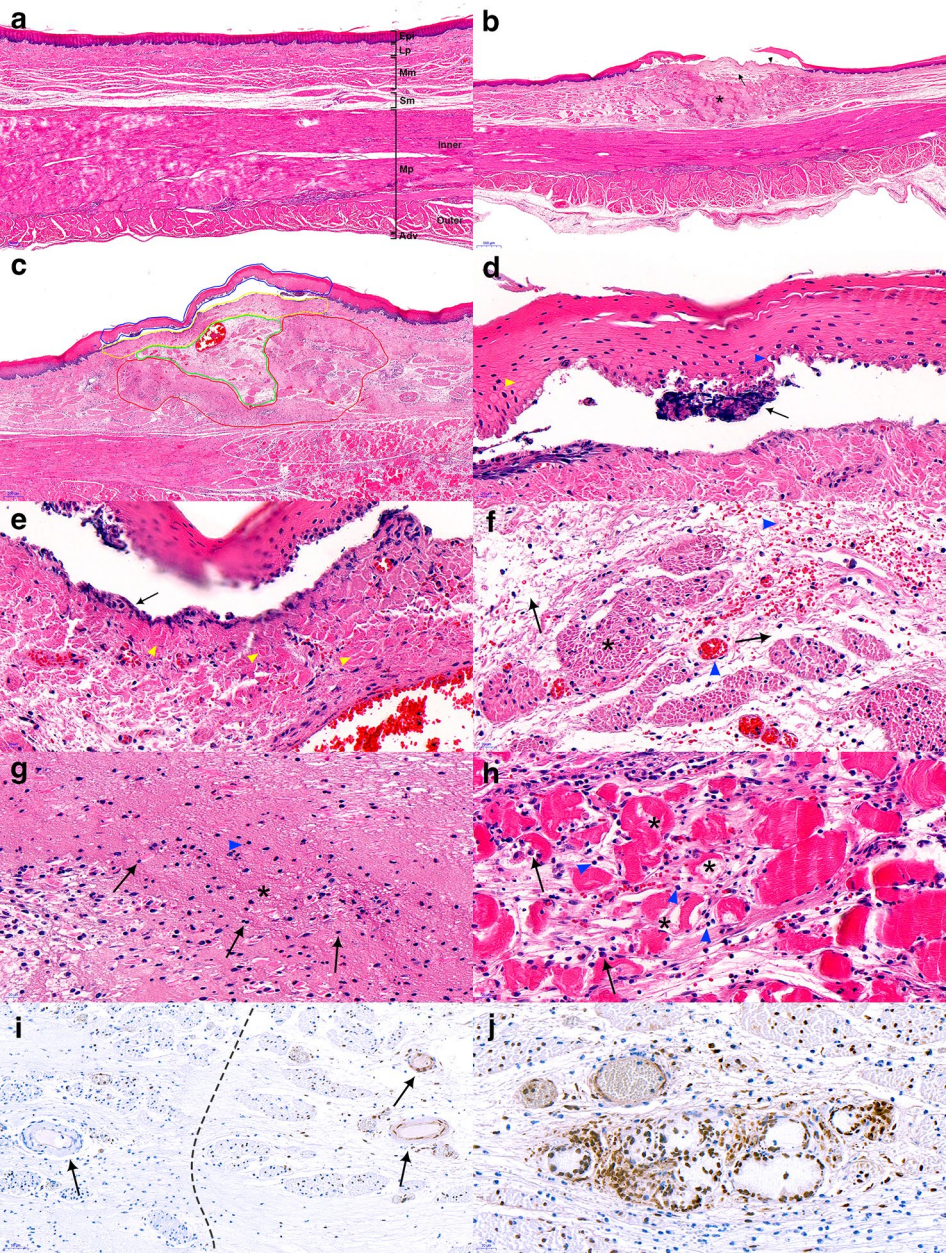
Histologic analysis of post-esophageal ablation tissue. (**a**) The normal esophageal wall shows a well-preserved morphology [40×, hematoxylin and eosin (H&E) staining]; (**b**) Ablated esophageal histology with 1500 V reveals edema and extensive submucosal necrosis (asterisk—necrosis, arrow—edema, arrowhead—epithelial detachment, 20×, H&E); (**c**) Ablation with 2000 V demarcates five compartments (blue line as zone 1, yellow lines as zone 2, green line as zone 3, red line as zone 4, and white line as zone 5); (**d**) Zone 1—detachment of the stratified squamous epithelium (yellow arrowhead—polygonal epithelium, blue arrowhead—edematous change, black arrow—clumped cells, × 400, H&E); (**e**) Zone 2—fibrotic lamina propria infiltrated by inflammatory cells (yellow arrowhead—dense fibrotic change, black arrowhead—karyolysis, 400 × , H&E); (**f**) Zone 3—edematous submucosa with congested blood vessels and degenerating smooth muscle cells (asterisk—degenerated muscularis mucosa, black arrow—edema, blue arrowhead—congestion with some hemorrhage, 400×, H&E); (**g**) Zone 4—extensive necrosis in the submucosa (asterisk—necrosis, black arrow—lymphocyte, blue arrowhead—small fragments of dead nuclei, karyorrhexis, 400×, H&E); (**h**) Zone 5—necrotic muscularis propria with inflammatory cells (asterisk—necrotic muscularis propria, black arrow—plasma cell, blue arrowhead—lymphocyte, 400×, H&E); (**i**) Vessels in the submucosal layer with terminal deoxynucleotidyl transferase (dUTP) nick end labeling (TUNEL) assay demarcating ablated and unablated areas (200×) with brown-stained nuclei (left, unablated; right, ablated); (**j**) Apoptotic submucosal gland with the maintenance of structural integrity (400×, TUNEL).

The non-ablated esophageal wall structurally maintained four layers of anatomy and histology: mucosa, submucosa, muscularis propria (inner circular skeletal muscle and outer longitudinal smooth muscle), and adventitia (Fig. 4a). Upon treatment with an electrical stimulus of 1500 V, the stratified squamous epithelium became detached with the destruction of the basal cell layer (Fig. 4b). The lamina propria and submucosa appeared pale due to edematous changes and was congested around the vessels. H&E staining for ablation at 2000 V is illustrated in Fig. 4c. The ablated tissues were swollen and thickened, similar to those subjected to 1500 V. Histological changes were characterized into five distinctive zones: zone 1, detached epithelium; zone 2, destruction of the basal layer; zone 3, pale area in the muscularis mucosa spanning the submucosal layer; zone 4, a dense pinkish area that encircles zone 3; and zone 5, edematous and disorganized muscularis propria layer.

Stratified squamous epithelium separated from the basement membrane was detected in zone 1. The degenerated epithelium featured small, condensed, and disintegrated nuclei; the epithelium near the basement membrane appeared to be more damaged than the superficial epithelium (Fig. 4d). The lamina propria in zone 2 consists of necrotic cells from the basal cell layer. The nuclei underwent karyolysis, and infiltration of nucleated immune cells was observed. The lamina propria layer itself was focally fibrotic and inflamed, whereas part of the remaining area revealed an edematous change (Fig. 4e). The region of interest chosen as zone 3 encompassed the muscularis mucosa and submucosa layers and was accompanied by edema with loose collagenous connective tissue, scattered lymphocytes, and red blood cells (RBCs). The vessels were dilated and congested with red RBCs. The muscularis mucosa layer underwent degeneration along with an expanded interstitial space (Fig. 4f). A dense pinkish area resembling stardust in the submucosa was a consequence of lysed and necrotizing cells. “The graves of cell” were created by small fragments of dead nuclei, lymphocytes, and fibrocytes being implicated together (Fig. 4g). Zone 5 reflects damaged muscularis propria, which was extensively disorganized and occupied by necrotic myocytes, as well as infiltrated lymphocytes and plasma cells (Fig. 4h). TUNEL staining outlined the border between the treated and untreated areas (Fig. 4i). The nuclei of vessels and smooth muscles in the ablated areas were stained brown. The overall submucosal gland retained its structure, and the lining cells of the gland stained brown appeared to undergo apoptosis (Fig. 4j).

#### Overall ablation results of a balloon-type IRE catheter on area, depth, and electrical parameters

In the present study, we completed 24 ablations in six pigs. Of the ablative trials, 12 ablations were investigated using a basket-type catheter, and the remaining were investigated using a balloon-type catheter. Table 1 summarizes the 12 successful ablations of the balloon-type catheter. The most frequently damaged layers at 1000 and 2000 V were the submucosa (66.7%) and muscularis propria (100%), respectively.

**Table 1:** Demographics and characteristics of the balloon-type irreversible electroporation (IRE) catheter.

Number #	Applied voltage (V)	Pre-IRE impedance (Z)	Post-IRE impedance (Z)	Average current (A)	Current density (A/mm^2^)	Electrical energy (J)	Damaged surface area (mm^2^)	Damaged layer (deepest)	Damaged depth (µm)
1	1500	4825	3656	5.8	0.4	1.9	104.6	SM2	369.5
2	1500	4636	3653	6.0	0.4	1.9	112.3	SM2	453.2
3	1500	4923	3665	3.6	0.2	1.8	108.7	SM2	498.9
4	1500	4288	3655	3.4	0.2	2.1	93.2	SM2	691.6
5	1500	4851	3663	4.5	0.3	1.9	101.9	SM2	416.9
6	1500	4913	3659	4.3	0.3	1.8	106.0	PM	837.3
7	2000	4714	251	7.9	0.5	3.4	149.1	PM	1167.4
8	2000	4812	262	8.1	0.5	3.3	138.6	PM	1157.4
9	2000	4557	270	8.3	0.6	3.5	141.2	PM	935.3
10	2000	4982	267	7.0	0.5	3.2	132.6	PM	865.0
11	2000	4685	222	9.9	0.7	3.4	140.3	PM	592.4
12	2000	4755	236	7.9	0.5	3.4	150.0	PM	730.2

*V* volt, *Z* impedance, *A* ampere, *J* Joule, *SM2* submucosal layer (half below), *PM* propria muscularis, *MM* muscularis mucosa.

**Figure 5:**
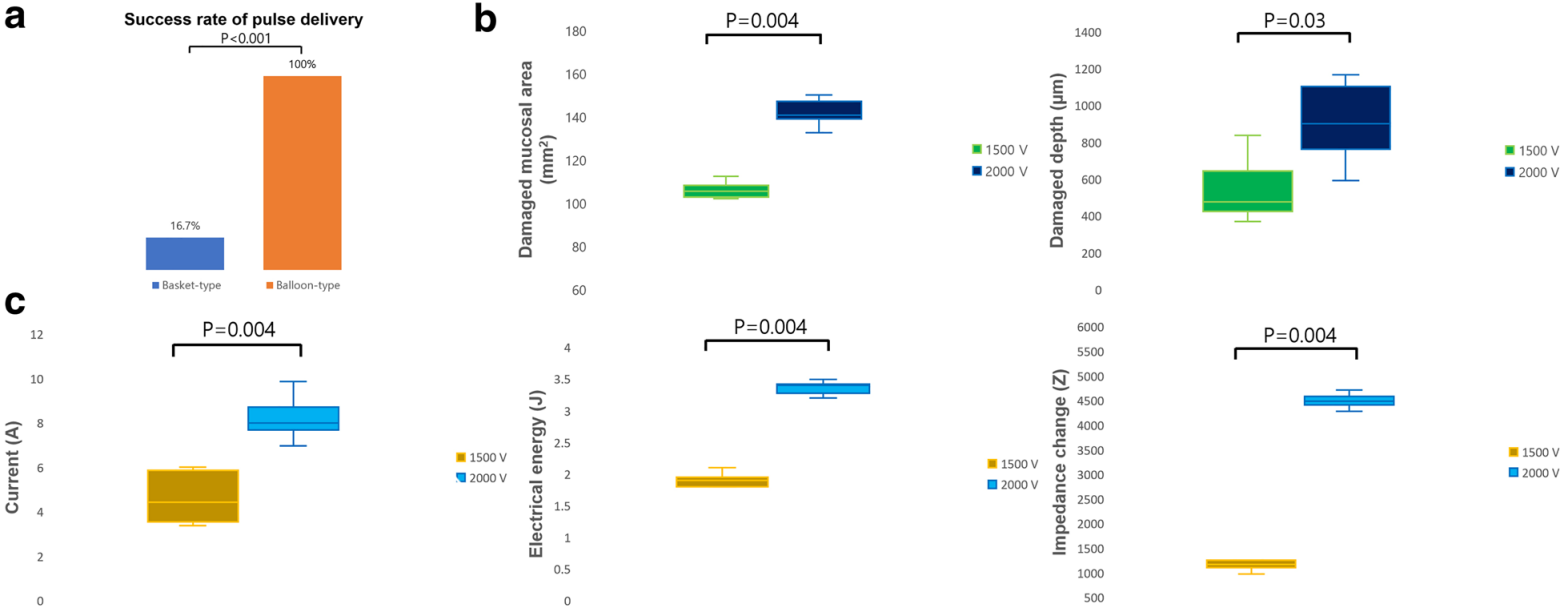
Comparison of 1500 V- and 2000 V-electroporated ablation using balloon-type catheters. (**a**) Success rate of consecutive 40-pulse delivery. Fisher’s exact test was used to examine categorical variables; (**b**) Irreversible electroporation ablation–induced damaged surface area (left) and depth (right); (**c**) Major electrical parameters measured during ablation (left, current; middle, electrical energy; right, impedance change). The Mann–Whitney test was used for the non-parametric analysis. Data are expressed as the median and interquartile range. *P*-value < 0.05 are considered significant.

As shown in Fig. 5 and Supplementary Table 1, the results stratified by electrical voltage were analyzed. The success rate at the primary endpoint showed a remarkable difference of 100% (12/12) for the balloon catheter and 16.7% (2/12) for the basket-type catheter (p < 0.001) (Fig. 5a). Gross inspection suggested a significant increase in the damaged surface area within the groups (1500 V; 105.3 mm^2^ vs 2000 V; 140.8 mm^2^, p = 0.004) (Fig. 5b). Furthermore, TUNEL positivity, which indicates the depth of ablation, was remarkably different between groups (476.1 μm vs. 900.1 μm, p = 0.03) (Fig. 5b). The measured current during 2000 V ablation was twice as high as that observed at 1500 V (4.4 A vs 8.0 A, p = 0.004). The median calculated electrical energy at 1500 V was 1.9 J, while that at 2000 V was 3.4 J. An impedance drop of 2000 V occurred significantly more often than that at 1500 V (1178.5 Z vs. 4491 Z, p = 0.004) (Fig. 5c).

## Discussion

Given the breadth and depth of knowledge of IRE, the academic understanding and clinical applications of this procedure have now expanded. IRE is a nonthermal ablation therapy that delivers a multiple pulse sequence to yield a cumulative effect, ultimately leading to tissue damage within the targeted region. We previously observed that it was challenging to identify whether the electrodes had achieved full contact for activation whenever esophageal spasms occurred during ablation through a basket-type catheter^13^. Moreover, frequent arching impedes the effective delivery of desired sequences. Therefore, the fundamental purpose of this study was to devise a catheter capable of precisely targeting sequences at the lesion of interest that would warrant successful IRE. The novelty of this study lies in that it is the first in vivo experiment to validate superiority of a balloon-type IRE catheter, validating its superiority in terms of its efficacy and safety compared to basket-type catheters.

Our study demonstrated the higher efficacy of balloon-type versus basket-type IRE catheters in the esophagus, along with changes in histopathology and electrical parameters of balloon-type catheters, depending on electrical energy (1500 V vs. 2000 V). All procedures using balloon-type catheters achieved full electrical pulse sequences administered to the esophagus, without acute complications. As electrical energy increased, the ablated esophagus revealed a damaged surface and increased depth, demonstrating the effect of tissue destruction. Regarding the electrical parameter analysis, increased current and decreased impedance occurred during IRE stimulation, which increased conductivity. Therefore, our findings of histological and electrical changes implying conductivity increases during pulse delivery suggest enhanced tissue permeability by cell membrane breakdown and temperature elevation due to Joule heating, especially at 2000 V (1274 V/cm). If we assume that apoptosis occurs due to IRE (> 350 mV/cm), the simulated ablation depth (approximately 500 µm; 1500 V, 1000 µm; 2000 V) was very similar to the actual histologic depth (median 476 µm; 1500 V, 900 µm; 2000 V) and the temperature surrounding the electrode was elevated within the estimated range (43 °C and 48 °C) (Supplementary Fig. 1). These results suggest that tissue damage was unaffected by the thermal effects during the procedure.

As aforementioned, our purpose of introducing IRE for the esophagus was to treat superficial esophageal cancer and dysplasia. Based on our histologic results, although the ablation depth post-IRE was significantly deeper at 2000 V compared to 1500 V, the ablation depth showed variability at each electrical voltage. The reason for the non-uniformity in ablation depth is likely related to the location of the ablation tissue. Esophageal tissue thickness varies depending on the location^19^. As tissue thickness increases, the depth of electroporation decreases while keeping electrical field intensity the same^20^. This may be due to increased electrical resistivity when the conductivity decreases, resulting in current flow to decrease. With the exception of tissue thickness, both non-standardized balloon pressure^21^ and tremor are also thought to have an effect on the ablation depth. Further improvements in IRE procedure will be necessary to achieve a reliable and consistent ablation depth in the esophagus. Balloon-type catheters, devised exclusively for this study, differ from basket-type catheters in terms of efficacy. The basket-type catheter is more prone to shortening and arching from frequent spasms owing to the decreased space between the flexible electrodes, while the balloon-type was rigid enough to withstand such muscle spasms and could deliver the scheduled sequences. Not only did it localize the ablation site, but the transparency of the balloon also made it possible to directly observe the target area. These distinct features of the balloon-type catheter enhance ablation accuracy at the target lesion and mitigate the migration of electrodes during muscle contractions.

In addition, based on previous experimental studies, the optimal spacing of electrodes for IRE to be performed on important structures at different temperature settings was documented to be 10–15 mm^22^. The radius of curvature of the inter-electrode adopted for our investigation was 16 mm, which is compatible with the recommended value for the induction of apoptosis without thermal damage. The electrodes contact area of the balloon-type IRE catheter used in this study exceeded the threshold and induced apoptosis, while the inter-electrode area did not fully achieve the electrical field intensity sufficient to induce apoptosis. Since the balloon-type catheter is still a prototype, ablation is required in all areas that include the two electrodes in order to achieve focal ablation therapy. In order to accomplish this, we recommend enhancements to enlarge the electrode area, reduce the inter-electrode distance, and minimize the thermal effect. Further studies are needed to deepen our knowledge and insights regarding mathematical modeling and electrode arrays.

Regarding the thermal implications, the onset of protein denaturation after thermal coagulation usually begins to develop at a temperature of 42 °C, whereas heat-induced shrinkage occurs at temperatures > 50 °C^23^. This investigation was primarily conducted based on COMSOL simulation data to exclude thermal effects. It was verified that the thermal effect was minimal for 4 s during ablation at voltages of 1500 and 2000 V, consistent with the IRE histological findings. Instead, IRE caused tissue apoptosis while tissue morphology and structure held intact, leading to tissue necrosis and destruction, as ascertained by a TUNEL assay.

Previous studies related to the histopathology of IRE ablation have reported the simultaneous features of both apoptosis and necrosis^24^. In this study, the overall tissue characteristics presented morphological changes, such as pyknosis, congestion, edema, and necrosis. These esophageal tissue changes following IRE ablation documented findings similar to those described in the study by Vogel et al., which described IRE ablation in pigs’ livers^25^. In particular, zones 3 and 4 in our study were compatible with the homogeneous zone, which indicates ischemic changes characterized by poor blood supply, swelling, and necrosis. Another study reported that IRE pulses can reduce blood flow in tissue hypoxia characterized by vascular congestion and further induce cell death^26,27^. These changes are known to subsequently induce dysfunction of the electron transport chain in the mitochondria, leading to decreased ATP production, and dysfunction of sodium–potassium and calcium pumps on the cell surface. The failure of ion pumps causes cell swelling owing to hyperosmolarity. Prolonged ischemia-related changes induced by IRE promote apoptosis, necrosis, and necroptosis^28^. Thus, our histological findings strengthen previous findings that IRE-induced tissue hypoxia causes further cell death^26^. However, little information is available in the literature regarding the effects of IRE on the esophagus. One report describing a paraesophageal IRE investigation performed on six rabbits suggested that the most effective IRE energy parameters were 1500 V/cm with 90 ms when the thermal energy from IRE was dissipated across the esophagus^29^. Another study on the esophagus of eight pigs substantiated the safety issues of IRE^30^. Although the aforementioned studies investigated esophageal luminal injury from repeated IRE on the adventitia, our study scrutinized whether the injury occurred on the muscularis propria layer, the most important layer for minimally invasive treatment of hollow viscera, owing to pain sensation. Regarding ablation of the epithelium, the delivered energy level (100–200 J) for creating myocardial injury was much larger than that for mucosal ablation (1.2–2.2 J) due to the electrode area and tissue properties.

In addition to IRE, RFA is a well-established thermal ablation therapy (70–90 °C) for esophageal tumor destruction. However, the heat sink effect adjacent to large tumors is a common obstacle to complete removal. According to a report from the United States RFA registry, Barrett’s disease has a recurrence rate that almost surpasses 25% following RFA eradication^31^. Considering the risk factors of neoplastic progression of Barrett’s esophagus, careful endoscopic surveillance with histologic interpretation following RFA is recommended. Another low-temperature cryoablation technique (Pentax Medical Inc., Japan), which induces cellular apoptosis through tissue freezing and thawing, has recently emerged. Although cryoablation is now gaining attention as a useful ablative method, its ablation time (median, 17 s; total time, 30 s) is longer than that of IRE, and the procedure is associated with the inconvenience of applying an additional cryogenic fluid spray system^32^. Further comparisons of the clinical efficacy and long-term oncological outcomes of the aforementioned ablation therapies would benefit clinicians.

This study highlights that endoscopy-assisted IRE ablation can be used to treat EC. More specifically, if IRE can adjust for the ablation depth by manipulating energy intensity, it would be feasible for use in mucosa-confined squamous carcinoma. Although effective in treating mucosal cancer, endoscopists usually experience difficulty performing esophageal ESD accompanying narrow lumen and moving wall, especially among older patients with comorbidities^33,34^. Furthermore, adverse events after esophageal ESD are significant when the resected area is so large that the muscle layer is exposed^35^. Because IRE is limited with regards to specimen acquisition and may not completely replace ESD, there is potential for IRE to serve as an adjuvant or monotherapy for T1a staged esophageal cancers such as flat type superficial esophageal squamous carcinoma or high grade intramucosal carcinoma.

A limitation of this study was that the actual measurement of luminal esophageal temperature could not be implemented, although an escalation of maximal temperature was expected. Several studies have monitored temperature using esophageal probes^36^. However, in our study, the catheter could not be configured to allow the temperature probe to advance together with the endoscope, making the real-time monitoring of temperature unattainable. Another weakness implicated in the porcine model used in this investigation was the use of a normal esophagus model. However, there is currently no established EC model of swine^37^, and our study primarily aimed to evaluate the efficacy of balloon-type catheters over basket-type catheters. Thus, narrow electrical energy uses for IRE seem to be allowable because this investigation is not focused on delicate energy ranges for EC. Although only two energy settings within non-thermal conditions (1500 V and 2000 V) were compared in our experiment, a more comprehensive long-term histologic study based on various electrical energy parameters would be essential to design a safer procedural protocol for IRE^29^. In our study, pigs were sacrificed 24 h after ablation for histological analysis. Although this time point was set to previous liver IRE study^38^, it would be necessary to establish an optimal time frame for the post-IRE assessment of the esophagus. Nevertheless, since the 24-h time point was consistently applied across all subjects in this study, it is unlikely to have a significant change on histological and safety assessment. Finally, the number of our experiments was based on statistical sample size calculations aiming at the efficacy of balloon-type catheters. Nonetheless, a larger sample size is needed to enhance the validity of the statistical power and to yield a more generalized result.

## Conclusion

We achieved the electrical conditions of nonthermal esophageal IRE resulting from the simulation. Over-the-wire balloon-type IRE catheters overcame intense esophageal muscle spasms during the procedure, in contrast to basket-type catheters. The former demonstrated excellent efficacy and histological safety at 1500 V (955.1 V/cm). Esophageal IRE at 2000 V (1273.9 V/cm) revealed necrosis and inflammation of the muscularis propria.

## Supplementary Information

Supplementary Information.

## Data Availability

All data generated or analyzed during this study are included in this published article and its supplementary information files.
