# Supplementary material for: Assessment of efficacy and safety of advanced endoscopic irreversible electroporation catheter in the esophagus
Source: Sci Rep. 2023 May 16;13:7917. doi: 10.1038/s41598-023-33635-9 (PMC10188560; doi:10.1038/s41598-023-33635-9)

Supplementary information

Supplementary table 1. Pathologic and electrical parameters of irreversible electroporation (IRE) with a balloon catheter based on the electrical field intensity.

Ablated area and depth	1500 V (N=6)	2000 V (N=6)	P-value
Damaged surface area (mm^2)	105.3 (99.7-109.6)	140.8 (137.1-149.3)	0.004†
Damaged layer (deepest)			0.02*
MM	1 (16.7)	0	
SM	4 (66.7)	0	
PM	1 (16.7)	6 (100)	
Damaged depth (μm)	476.1 (405.1-728.0)	900.1 (695.7-1159.9)	0.03†
**Electrical parameters**	**1500 V (N=6)**	**2000 V (N=6)**	**P-value**
Average current (A)	4.4 (3.5-5.9)	8.0 (7.7-8.7)	0.004†
Impedance change (Z)	1178.5 (1113.5-1255.0)	4491.0 (4419.0-4591.3)	0.004†
Electrical energy (J)	1.9 (1.8-2.0)	3.4 (3.3-3.4)	0.004†

SM, submucosal layer; PM, propria muscularis; MM, muscularis mucosa; TUNEL, terminal deoxynucleotidyl transferase dUTP nick end labeling, volt; A, ampere; J, Joule

*, Fisher exact method for categorical variables.

†, Mann-Whitney test for non-parametric analysis. Data was expressed as median and interquartile range (IQR).

P < 0.05 is considered to be statistically significant.

Supplementary figure 1. Simulation of electric field intensity and thermal distribution after 40 pulses. Electrical field intensity at (a) 1500V and (b) 2000V, and thermal distribution at (c) 1500V and (d) 2000V.

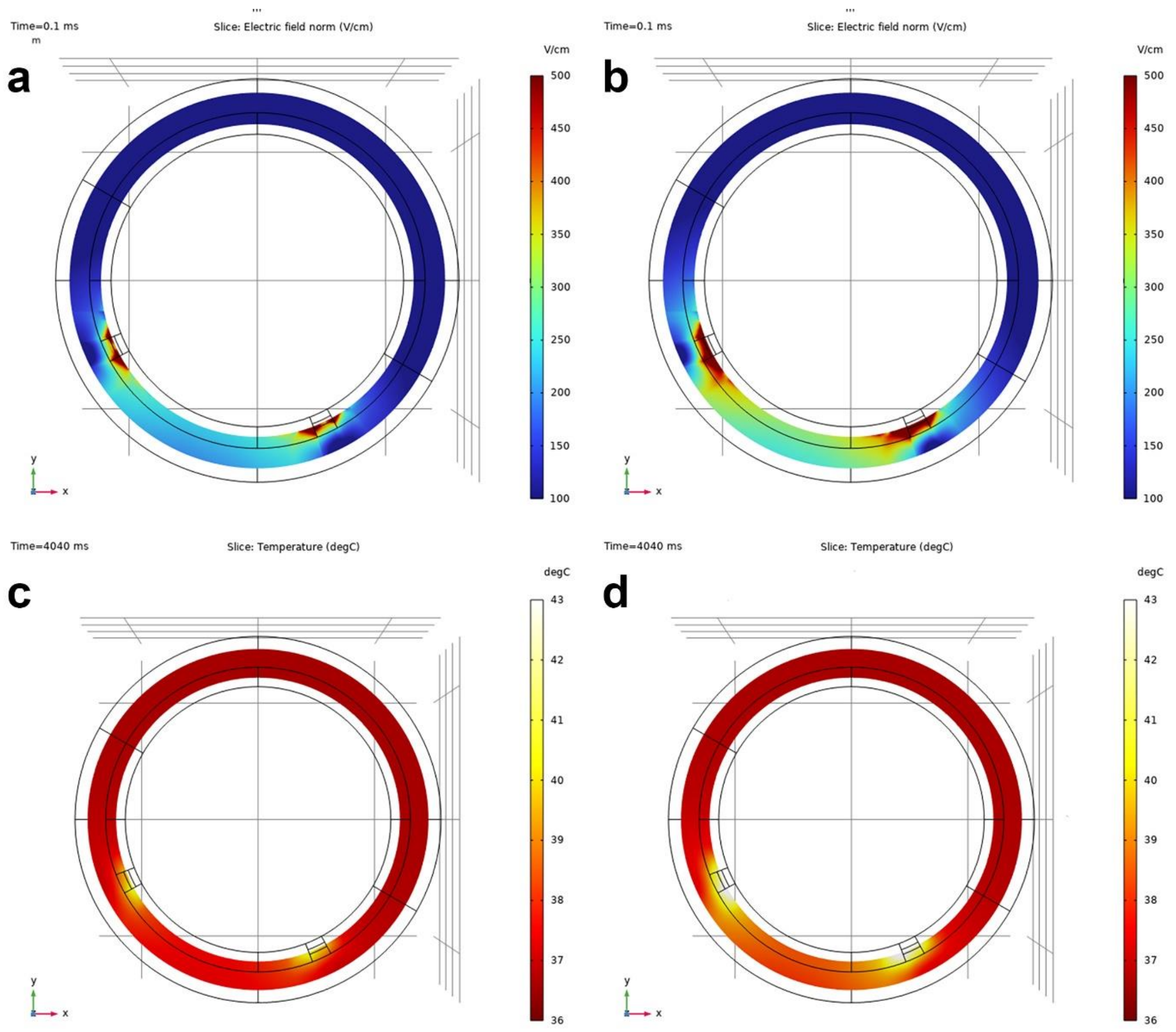

Supplement: Supplementary file 1 — Supplementary Information. [file 41598_2023_33635_MOESM1_ESM.pdf]
